# Image harmonization improves consistency of intra-rater delineations of MS lesions in heterogeneous MRI

**DOI:** 10.1016/j.ynirp.2024.100195

**Published:** 2024-02-02

**Authors:** Aaron Carass, Danielle Greenman, Blake E. Dewey, Peter A. Calabresi, Jerry L. Prince, Dzung L. Pham

**Affiliations:** a Department of Electrical and Computer Engineering, Johns Hopkins University, Baltimore, MD, 21218, USA; b Center for Neuroscience and Regenerative Medicine, The Henry M. Jackson Foundation for the Advancement of Military Medicine, Bethesda, MD, 20817, USA; c Department of Neurology, Johns Hopkins School of Medicine, Baltimore, MD, 21287, USA; d Department of Radiology, Uniformed Services University of the Health Sciences, Bethesda, MD, 20814, USA

**Keywords:** Harmonization, Magnetic resonance imaging, Multiple sclerosis, White matter lesion, Lesion segmentation

## Abstract

Clinical magnetic resonance images (MRIs) lack a standard intensity scale due to differences in scanner hardware and the pulse sequences used to acquire the images. When MRIs are used for quantification, as in the evaluation of white matter lesions (WMLs) in multiple sclerosis, this lack of intensity standardization becomes a critical problem affecting both the staging and tracking of the disease and its treatment. This paper presents a study of harmonization on WML segmentation consistency, which is evaluated using an object detection classification scheme that incorporates manual delineations from both the original and harmonized MRIs. A cohort of ten people scanned on two different imaging platforms was studied. An expert rater, blinded to the image source, manually delineated WMLs on images from both scanners before and after harmonization. It was found that there is closer agreement in both global and per-lesion WML volume and spatial distribution after harmonization, demonstrating the importance of image harmonization prior to the creation of manual delineations. These results could lead to better truth models in both the development and evaluation of automated lesion segmentation algorithms.

## Highlights

• Demonstrate the benefits of image harmonization on the consistency of white matter lesion segmentation/parcellation.
• Introduce a simplified scheme to understand intra-rater and inter-rater consistency on binary object detection tasks.
• Highlight that manual delineations should no longer be created on imaging data that is not harmonized.

## 1 Introduction

Magnetic resonance imaging (MRI) is a widely used imaging modality that employs strong magnetic fields, magnetic field gradients, and radio-frequency signals to generate images of the soft tissues of the body (Hoult and Bahkar, 1998; Hornak, 2006). MR scanners can be configured both in hardware and software to acquire images with many diverse tissue contrasts, providing great flexibility for both clinical and research purposes (Sudhyadhom et al., 2009). This very flexibility, however, has made it extremely challenging—and practically impossible at present—to develop a standardized intensity scale that can be reproduced between scanners (Nyúl and Udupa, 1999; Zuo et al., 2023b). As a result, even when the tissue contrast is specified to be broadly the same, e.g., a *T*_1_-weighted (*T*_1_ − *w*) image, the resulting images from different scanners or at different times on the same scanner will, inevitably, be somewhat different in their intensity scales (Han et al., 2006). These differences, which are generally not correctable by simple intensity scaling, can lead to different interpretations of the anatomy within the images, depending on the subjectivity of the viewer.

There is an emerging collection of methods to address the issue of MR intensity variations across scans, known as “*MR harmonization*” (Dewey et al., 2018; Garcia-Dias et al., 2020; Guan et al., 2021; Mirzaalian et al., 2016; Zuo et al., 2022, 2023b, 2023a); which can be broken down into the two broad categories of “*image-level MR harmonization*” and “*statistical MR harmonization*”. Within both frameworks, scanners are often viewed as domains, where it it understood that the anatomy will be imaged differently in different domains. Image-level MR harmonization takes the approach of adjusting the image, not its underlying anatomy (Bashyam et al., 2022; Cackowski et al., 2023; Dewey et al., 2019; Dinsdale et al., 2021; Guan et al., 2021; Torbati et al., 2021; Zuo et al., 2021a,b, 2022, 2023b,a). These approaches have their roots in MR synthesis, which is the practice of generating synthetic MR images from one or more source images (Hofmann et al., 2008, 2011; Roy et al., 2013). These intensity transformations can be learned in a variety of ways (Burgos et al., 2014, 2015; Chartsias et al., 2018; Duchateau et al., 2018; Hofmann et al., 2008, 2011; Han, 2017; Huang et al., 2018; Jog et al., 2017; Joyce et al., 2017; Lee et al., 2017; Liu et al., 2023; Roy et al., 2017; Zhao et al., 2017; Zuo et al., 2020), with recent work being focused on deep learning approaches (Hiasa et al., 2018; Liu et al., 2020; Sharma and Hamarneh, 2020; Yang et al., 2020; Zhou et al., 2020). Much work in MR image synthesis focuses on image translation tasks such as the generation of missing *T*_2_-weighted (*T*_2_ − *w*) images from observed *T*_1_ − *w* images (Rousseau, 2008). In contrast, image-level harmonization focuses on creating replacement images with a consistent intensity profile. For example, a set of images from one domain (i.e. site or scanner) that includes a *T*_1_ − *w* image might be used to synthesize a *T*_1_ − *w* image that looks as though it came from a different domain.

In contrast, statistical MR harmonization frameworks are used to compensate for the numerical differences that are produced by a single algorithm used on images from multiple domains (i.e. sites or scanners) (Fortin et al., 2017; Garcia-Dias et al., 2020; Pomponio et al., 2020; Zhu et al., 2019). They operate on image derived measurements like regional volumes (Zhu et al., 2019), which are generated from some segmentation algorithm (Fischl, 2012; Huo et al., 2016) and are dependent on the segmentation algorithm's performance. Given these measurements, the statistical-based harmonization approaches act as a post-processing step, aimed at correcting the variations within a segmentation method caused by intensity differences.

Both MR harmonization approaches are useful and have advantages in certain situations. Image-level MR harmonization generally requires training examples from multiple domains and their use can be limited to those they have been trained on. However once trained, these domain-adaptive algorithms can be used on individual scans. The statistical methods apply only to populations, and they learn to compensate for scanner differences from the data themselves and do not require training in advance. A key limitation of statistical MR harmonization is that it cannot produce modified images that can be viewed as if they come from a single scanner despite their origin. The ability to generate images is required if human raters are to be used to consistently label images from multiple sites—whether to generate truth models to train machine learning algorithms or to produce volumetric analyses for clinical or scientific purposes.

There has been considerable literature demonstrating the advantages of image-level harmonization to improve the consistency of automatic algorithms (Cackowski et al., 2023; Dinsdale et al., 2021; Guan et al., 2021; Hays et al., 2022; Zuo et al., 2021a; Shao et al., 2022); our goal in the present work is to demonstrate that harmonization is essential for consistent manual labeling. Work by Bozsik et al. (2022) notes that the inconsistency of manual delineators has a spatial bias to it, with the lesions in the optic nerve region being the most difficult to reliably identify. We focus on multiple sclerosis (MS) lesion segmentation (Dong et al., 2017; Elliott et al., 2013, 2014; Fleishman et al., 2017; Harmouche et al., 2015; Liu et al., 2022; Shiee et al., 2010; Tohidi et al., 2022; Zhang et al., 2019, 2024), as it is difficult to achieve intra-rater and inter-rater consistency, (Carass et al., 2017; Commowick et al., 2018, 2021; Styner et al., 2008) and has therefore been previously unreliable as a gold standard for establishing truth models for machine learning and scientific use. Properties derived from lesion segmentation such as lesion load, whether derived manually or automatically, have also proven to lack clinical utility in staging MS and evaluating utility of disease modifying therapies for MS (Barnett et al., 2021; McGinley et al., 2021; Tavazzi et al., 2020). Outside of MR harmonization there have been other efforts to standardize the MR acquisition process (Vrenken et al., 2013; Filippi et al., 2016; Stefano et al., 2022). There are various reasons why these standardized protocols are not fully embraced by clinicians, as the standards may differ from local preferences and best practices. In addition, scanner time is limited and expensive which imposes another constraint on what is feasible in a clinical environment. From a practical point of view these MR standardization efforts can only benefit future acquisitions. Whereas MR harmonization as a post-processing step can remediate past, present, and future acquisitions.

MS is a disease of the central nervous system that is characterized by inflammation and neuroaxonal degeneration in both gray matter (GM) and white matter (WM) (Compston and Coles, 2008; Reich et al., 2018). The demyelination process and reactive gliosis, which are defining characteristics of MS, can be quantified through MRI of the brain and spinal cord. This quantification is achieved through the use of the fluid-attenuated inversion recovery (FLAIR) MR imaging sequence; it is a sequence that produces strong *T*_2_ − *w* weighting, suppresses the cerebrospinal fluid (CSF) signal (Kates et al., 1996), and as such enables the detection of *T*_2_ − *w* lesions within the WM (or WMLs). WMLs have a hyperintense appearance on *T*_2_ − *w* MRI and have become a standard part of the diagnostic criteria (Thompson et al., 2018). Thus we are interested in understanding how the appearance of the *T*_2_ − *w* MRI influences the consistent identification of WMLs. Cohen et al. (2020) notes that differences in the identification and measurement of WMLs can arise from different scanners, acquisition protocols, image quality, and interpretation. They also note that use of structured radiological reports to quantify active lesions, lesion burden, and other measures do not overcome these differences. Efforts to strictly control the imaging platform—i.e., acquisition standardization—do not completely resolve these issues either (Mowry et al., 2020). It is our hypothesis that image-level harmonization can improve consistency in manual assessment of WMLs in *T*_2_ − *w* MRI.

In this paper, we explore intra-rater inter-scanner consistency and identifiability of WMLs both with and without harmonization. We use a cohort of ten subjects imaged on two different scanners and have created harmonized versions of these scans using DeepHarmony (Dewey et al., 2019). A human rater manually identified the WMLs on these sets of scans—structural images from both scanners and structural harmonized images for both scanners. This was done independently for each subject and the rater was blinded to the source of the data (i.e., original or harmonized). To compare these set's of delineations we expand upon an object detection classification framework for this task. In Sec. 2 we outline this previously developed object detection classification scheme and modify it for our scenario in Sec. 3. Our analysis of our intra-rater inter-scanner comparison is described in Sec. 4 and includes both subject and object detection classification level comparisons.

## 2 Background

**Fig. 1:**
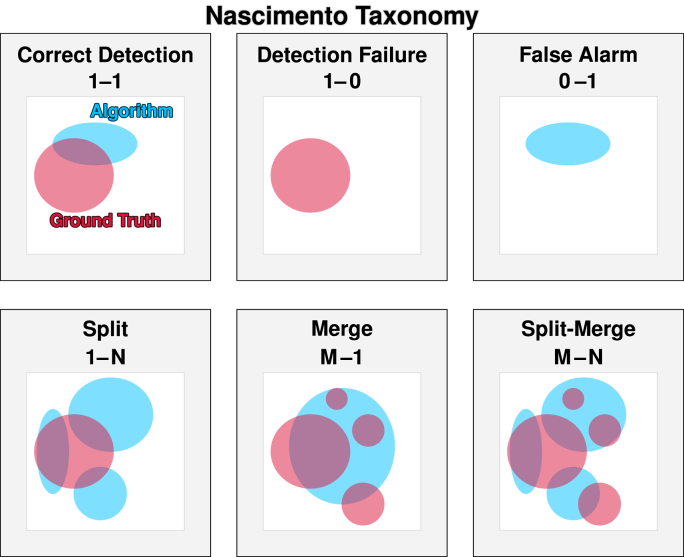
Illustrated are the six classes in the Nascimento Taxonomy. Each panel includes the name for the specific class and also features the notation *X*–*Y* denoting the number of components from the ground truth, *X*, and the automated algorithm, *Y*. For example the “Correct Detection” class has the notation 1–1, as there is a one-to-one correspondence between the ground truth identified object and automated algorithm object. See Sec. 2 for a description of all six classes.

In order to compare intra-rater delineations on either original or harmonized images, we adopt the work of Nascimento and Marques (2006) for classifying object correspondences in segmentations. Their nomenclature described the classification of various types of matches that can occur between segmentations involving a ground truth from an expert and a second segmentation from an automated algorithm. The complete taxonomy of their classification forms six classes, described below, with illustrations of each class in Fig. 1. We will first outline this object detection classification framework, before we modify it to suit our needs of comparing two manual delineations, both of which are considered the *ground truth*.

Given two segmentations, one ground truth and the other from an automated algorithm, we can consider how the connected components of these two segmentations relate to each other. We want to know if given a manually identified object, did the algorithm identify a single corresponding connected component that uniquely overlaps with the manual segmentation? If yes, such an object is said to have been a “Correct Detection” and we note that the ground truth and algorithm identified objects are in one-to-one correspondence (1–1). It is straightforward to identify two other classes: the case of “Detection Failure” in which the ground truth has identified an object but the algorithm has no corresponding object—one-to-zero correspondence (1–0); and the case of “False Alarm” occurs when the algorithm has identified an object that has no corresponding ground truth—zero-to-one correspondence (0–1). The “Detection Failure” and “False Alarm” classes can be thought of as the antithesis of each other. These three classes are depicted in the top row of Fig. 1.

The first of the remaining three classes in the Nascimento Taxonomy, arises from the case where a ground truth object overlaps with multiple objects from the automated algorithm and those objects do not overlap with anything else. In this case the ground truth object has been split up into multiple different objects by the algorithm. We naturally refer to this as the “Split” class and note that the objects are in a one-to-many correspondence (1–N). Similar to the “Detection Failure” and “False Alarm” classes, the antithesis of the “Split” class is the “Merge” class which has a many-to-one correspondence (M–1). In this class, many ground truth objects overlap with a single automated algorithm object. Finally, we identify the “Split-Merge” class which has a many-to-many correspondence (M–N). In this class, both the ground truth and algorithm agree that there are a collection of objects, but the exact number of objects and their boundaries are not agreed upon. These last three classes are illustrated in the bottom row of Fig. 1. We observe that these six classifications represent a complete taxonomy for all possible overlap combinations between two segmentations. The Nascimento Taxonomy has been previously used to explore WML segmentation, applied on a per-lesion basis (Oguz et al., 2017; Carass et al., 2020). Those works explored the comparison between human raters and automated algorithms, using the ground truth from the human raters to distinguish the behavior of algorithms that reported similar global overlap-measures. We next modify the Nascimento Taxonomy to address our situation, in which we have two manual delineations, both of which are considered the *ground truth*.

## 3 Methods

### 3.1 Condensed Nascimento Taxonomy

The Nascimento Taxonomy is predicated on the concept of their being a unique ground truth against which algorithms can be compared. It provides a powerful tool for describing the specific performance behavior of various algorithms. However, it neglects an important subset of object detection analyses: The case of multiple ground truths or rather multiple manual delineations. It is very common, in all manner of problems, for multiple “ground truths” (or delineations) to exist. This is sometimes a result of multiple different raters with the goal of combining the delineations to form a “gold standard ground truth”, or to evaluate the intra-rater consistency of a specific delineator. In the latter case, it can be useful to know if the variability of a delineator with themselves is greater than that of a delineator with an algorithm. The key issue in both these cases, however, is the lack of a true ground truth for comparison. We now go through the six classes in the Nascimento Taxonomy, identifying the issues and propose a straightforward modification to address its use for the case of two manual delineations.

The first class, “Correct Detection”, in the Nascimento Taxonomy when applied to the case of two manual delineations can be readily interpreted as agreement between those delineations. We also note that in this situation the two manual delineations are in one-to-one correspondence (1–1). The “Detection Failure” class represents a situation in which the two manual delineations disagree about the presence of an object. The “False Alarm” presents a similar situation, when considering two manual delineations, in that the two delineations disagree about the presence of an object. When considering two manual delineations the “Split”, “Merge”, and “Split-Merge” classes all represent a situation in which the two delineations agree that there are some number of objects in a region but the boundaries and extents of those objects are not agreed upon.

We can now outline our Condensed Nascimento Taxonomy (CNT). Consider two human rater delineations G and H, with $G=\left\{g_{i}|i\in \left\{1,\ldots ,I\right\}\right\}$, $H=\left\{h_{j}|j\in \left\{1,\ldots ,J\right\}\right\}$, and *g*_*i*_ and *h*_*j*_ being the connected components of G and H, respectively. We define the cardinality of intersection operator, $\left|\cap_{X}\left(y\right)\right|$, as the number of connected components that the object *y* intersects with in the collection of connected components X, where X would correspond to a delineation (G or H) and *y* is an object from the other delineation. See Table 1 for a summary of this notation and corresponding definitions.

**Table 1:** A summary of the notation used in Sec. 3.1.

Notation	Meaning
G, H	Human rater delineations
*g*, *g*_*i*_	Respectively, a lesion or *i*th lesion of G
*h*, *h*_*j*_	Respectively, a lesion or *j*th lesion of H
*I*	Cardinality of G (i.e. object count in G)
*J*	Cardinality of H (i.e. object count in H)
$\cap_{X}\left(y\right)$	The set of lesions from the delineation X that intersect the lesion *y*; with *y* coming from the delineation Y
$\left|\cap_{X}\left(y\right)\right|$	Cardinality of $\cap_{X}\left(y\right)$ (i.e. number of objects in the delineation X that intersect with the object *y* from the delineation Y)

**Table 2:** Scanner and protocol specifications for our cohort of ten people with MS (PwMS). The *T*_1_ − *w* scans consisted of magnetization prepared rapid gradient echo (MPRAGE) and multi-echo magnetization prepared rapid gradient echo (MEMPRAGE). The FLAIR and PD-/*T*_2_ − *w* scans consisted of either 2D turbo spin echo (2D TSE) or 3D volume isotropic turbo spin echo acquisitions (3D VISTA). As appropriate, the echo times (TE), repetition times (TR), and inversion times (TI) are listed for each sequence. See Fig. 3 for example images of the *T*_1_ − *w* and FLAIR acquisition.

	Scanner #1	Scanner #2
**Scanner Hardware**	Philips Achieva 3T	Philips Achieva 3T
**Scanner Software**	R3.2.3	R5.1.7
**Receive Coil**	16 Channel Neurovascular	32 Channel Head
*T*_1_ − *w*	MPRAGE	MEMPRAGE
	1.1 × 1.1 × 1.18 mm	1 × 1 × 1 mm
	TE = 6 m, TR = 3 s, TI = 840 m	TE = 6.2 m, TR = 2.5 s, TI = 900 m
FLAIR	2D TSE	3D VISTA
	0.83 × 0.83 × 2.2 mm	1 × 1 × 1 mm
	TE = 68 m, TR = 11 s, TI = 2.8 s	TE = 125 m, TR = 4.8 s, TI = 1.6 s
PD-/*T*_2_ − *w*	2D TSE	2D TSE
	1.1 × 1.1 × 2.2 mm	1 × 1 × 3 mm
	TE = 12 m/80 m, TR = 4.2 s	TE = 11 m/100 m, TR = 3.4 s

Then, we define the “Agreement” class of the CNT as those *g*'s with $|\cap_{H}\left(g\right)|=1$ with a corresponding *h*′ such that *g* ∩ *h*′ ≠ ∅ and $|\cap_{G}\left(h^{\prime }\right)|=1$, and equivalently those *h*'s with $|\cap_{G}\left(h\right)|=1$ with a corresponding *g*′ such that *g*′ ∩ *h* ≠ ∅ and $|\cap_{H}\left(g^{\prime }\right)|=1$. This class is equivalent to the “Correct Detection” class in the Nascimento Taxonomy and equates to objects that are in a one-to-one correspondence. The Agreement class is made up of those objects (*g*'s and *h*'s) that are in one-to-one correspondence. We define a “Disagreement” class for the CNT as those *g*'s with $|\cap_{H}\left(g\right)|=0$ and those *h*'s with $|\cap_{G}\left(h\right)|=0$. This class is a colligation of the “Detection Failure” and “False Alarm” classes, corresponding to the one-to-zero and zero-to-one occurrences from the Nascimento Taxonomy.

Finally we have the “Checkered” class, which includes those g∈G with $|\cap_{H}\left(g\right)|>1$ and h∈H with $|\cap_{G}\left(h\right)|>1$. Clearly, these *g*'s and *h*'s have an overlap—thus, are not in the Disagreement class—but do not uniquely overlap a single object—thus are not in the Agreement class. The remaining objects that belong in the Checkered class are those *g*'s and *h*'s that have an overlap but the object they overlap does not uniquely overlap with them. We can write this out as those g∈G with $|\cap_{H}\left(g\right)|=1$ where the *h* with *g* ∩ *h* ≠ ∅ has $|\cap_{G}\left(h\right)|>1$. There is an equivalent definition for the *h*'s.

**Fig. 2:**
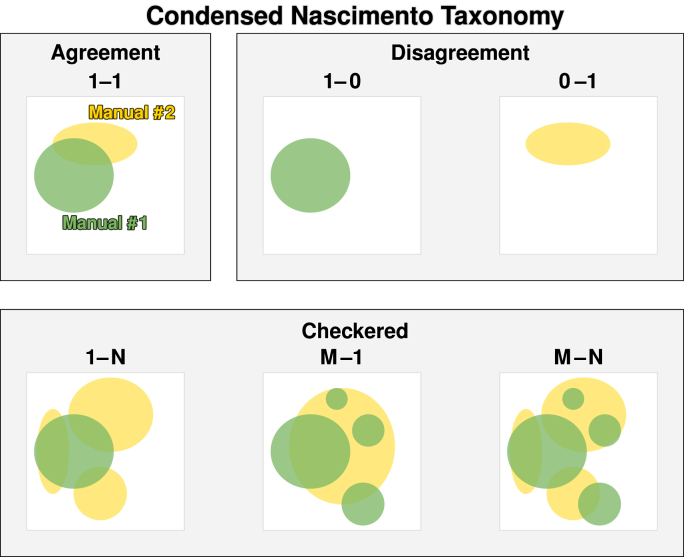
Illustrated are the three classes in the Condensed Nascimento Taxonomy (CNT). Each panel includes the name for the specific class and also features example cases for that class as well as notation *X*–*Y* denoting the number of components from each of the manual ratings. The panels include the same cases shown in Fig. 1 as they are classified according to the CNT. See Sec. 3 for a description of all the CNT classes.

There are two conceptual ways to prove that the CNT is a complete taxonomy of all possible object overlap combinations between two manual delineations. The first, and most obvious, is that every class of the Nascimento Taxonomy uniquely maps to a class of the CNT, and as the Nascimento Taxonomy is a complete taxonomy, the CNT must also be a complete taxonomy. The second, and almost certainly as obvious: consider any object g∈G, then $\left|\cap_{H}\left(g\right)\right|$ can only take the values 0, 1, or greater than 1. For the first and third case, that *g* would be assigned to the Disagreement and Checkered classes, respectively. In the case of $|\cap_{H}\left(g\right)|=1$, we simply need to check if the *h* with *g* ∩ *h* ≠ ∅ has any other overlaps. If $|\cap_{G}\left(h\right)|=1$, then *g* belongs to the Agreement class, otherwise $|\cap_{G}\left(h\right)|>1$ and *g* belongs to the Checkered class. Thus every g∈G is uniquely mapped to one of the three classes, similarly every h∈H is uniquely mapped to one of the three classes. The three classes of the CNT are illustrated in Fig. 2. In our use of the CNT, we use the Sørensen-Dice index (SDI) introduced by Dice (1945) and Sørensen (1948) as the sole measure of the degree of overlap between two segmentations. For the sets *A* and *B* we define the SDI as,

(1) $$\text{SDI}\left(A,B\right)=\frac{2|A\cap B|}{\left|A|+|B\right|},$$

where |⋅| denotes cardinality. The SDI returns values in the range [0, 1] with 1 meaning a perfect overlap between *A* and *B*, while 0 denotes no overlap between *A* and *B*. We note that there has been previous work (Wack et al., 2012) to improve delineation consistency and assessment. Wack et al. (2012) proposed two new tools for scoring the quality of a delineation in comparison to some known truth. In contrast, our work uses existing tools (SDI) while modifying the Nascimento Taxonomy to better represent our scenario.

**Fig. 3:**
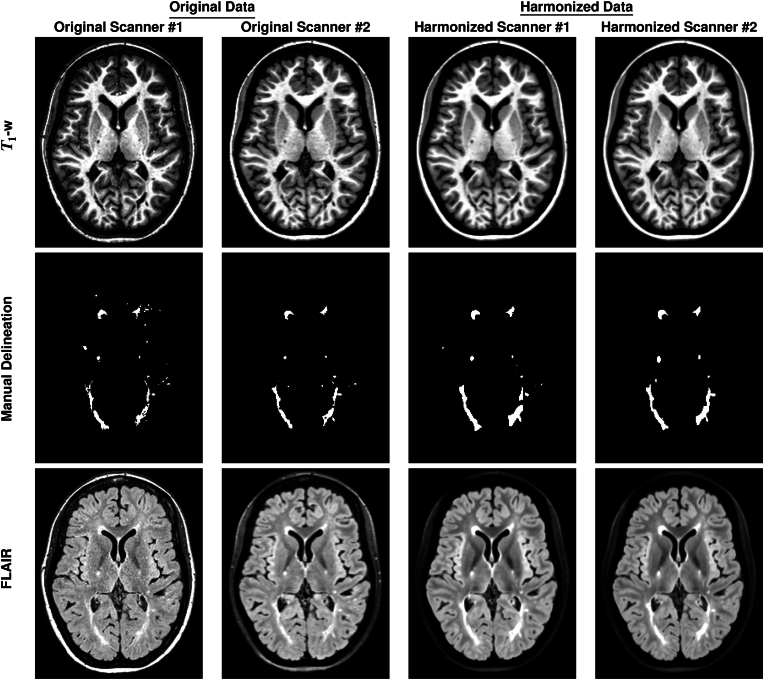
Shown are example images for one subject on both scanners, original and harmonized; as well as the corresponding manual masks. From left to right the columns are: Original Scanner #1, Original Scanner #2, Harmonized Scanner #1, and Harmonized Scanner #2. From top to bottom the rows are: *T*_1_ − *w*, Manual Delineation, and FLAIR. The *T*_1_ − *w* images are either MPRAGE or MEMPRAGE, see Table 2 and Sec. 3.2 for the complete details. The SDI for the original data for this subject is 0.572 and for the harmonized data is 0.795. For a different subject, we show a magnified portion of the FLAIR images and corresponding delineations in Fig. 4.

**Fig. 4:**
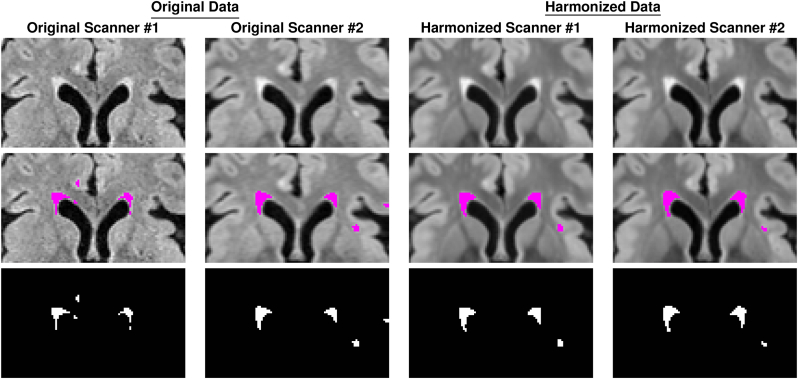
Shown in the top row is a zoomed portion of the FLAIR data from the Original Scanner #1, Original Scanner #2, Harmonized Scanner #1, and Harmonized Scanner #2. The bottom row is the corresponding manual delineations for the images in the top row. The center row is the FLAIR images (top row) overlaid with the manual masks (bottom row) colored in magenta.

### 3.2 Data & harmonization

Ten people with multiple sclerosis (PwMS) were scanned twice within 30 days on two separate Philips Achieva 3T scanners (Philips Healthcare, Best, The Netherlands) with differing head coils and different scanning protocols. The parameters for each scan are listed in Table 2. Each scan session included a set of standard structural images with a 3D *T*_1_ − *w* image, a 2D or 3D *T*_2_ − *w* FLAIR (fluid attenuated inversion recovery) image and a 2D turbo spin echo (TSE) sequence consisting of proton density (PD) and *T*_2_ − *w* images. See Fig. 3 for example images of the *T*_1_ − *w*, FLAIR, and the corresponding manual delineation. See Fig. 4 for magnified FLAIR images of a different subject, with the corresponding manual delineations.

Prior to harmonization the scans underwent the following preprocessing steps: bias-field correction (Tustison et al., 2010), super-resolution of the 2D scans to isotropic voxel sizes—3D scans were interpolated to isotropic voxel sizes—and anti-aliased (Zhao et al., 2018), rigid registration of all images with the *T*_1_ − *w* image of Scanner #2 (Avants et al., 2008), a second rigid registration to improve the alignment by incorporating the brain mask (Iglesias et al., 2011), and finally white-matter peak normalized (Reinhold et al., 2019) using the brain mask. See Dewey et al. (2019) for complete details of this preprocessing. We then used DeepHarmony (Dewey et al., 2019) to harmonize the images with the target contrast being that of Scanner #2; see Dewey et al. (2019) for complete details about the training of DeepHarmony. Our choice of DeepHarmony is motivated by its ability to harmonize the contrasts that are primarily used to delineate WMLs (i.e., *T*_1_ − *w*, FLAIR, and PD-/*T*_2_ − *w*). We used a previously published protocol (Carass et al., 2017) with some minor updates to complete the delineations. The complete details of our modified protocol can be found in Appendix A. In brief, the delineations were completed on the image volumes with the delineator reviewing both the *T*_1_ − *w* and FLAIR images simultaneously in the MIPAV software.^1^ The *T*_1_ − *w* and FLAIR pairs were provided in a randomized order, so the delineator did not know the source (i.e. original or harmonized) and the delineator saw multiple different subjects before seeing the same subject from a different source.

## 4 Results

At the most basic level we want to know if the lesion load or total number of lesions in a subject is influenced by whether the delineations are done on the original or the harmonized data. We refer to this as a *subject level analysis* and we detail it in Sec. 4.1. There are two goals in performing this basic comparison. First, we want to know if the harmonization has any impact on the delineations of the rater. If the rater is truly interpreting the images consistently then the harmonization will have a nominal effect on the delineations. If however, there are major differences between the delineations, then the harmonization is having some influence on how the MRIs are being read. The second goal, is more straightforward and simply wishes to establish if the rater delineations are at the consistency of previously reported intra-rater levels or inter-rater levels, or in the worst case not commensurate with either.

After the subject level analysis, we present a *lesion and class level analysis* to help refine our understanding of the rater's interpretations of the original and harmonized data. The results are in Sec. 4.2. In both of these analyses, we are interested in knowing if the lesion identification in the original data is different from identification on the harmonized data. When considering the original data, we compare the manual delineations of Original Scanner #1 (OS1) with the manual delineations of Original Scanner #2 (OS2). For the harmonized data, we compare the manual delineations of Harmonized Scanner #1 (HS1) with the manual delineations of Harmonized Scanner #2 (HS2).

### 4.1 Subject level analysis

By subject level analysis, we are interested in the SDI between the manual delineations for two comparisons: 1) original data and 2) harmonized data. The original data comparison measures the amount of agreement between the manual delineations between the OS1 and OS2 data. For the harmonized data we compare the manual delineations as done by looking at the HS1 data against the manual delineations based on the HS2 data. In Table 3 we report the range, median, mean, and standard deviation, for the SDI in our subject level analysis, across the ten PwMS. A two-sided paired Wilcoxon signed rank sum test (Wilcoxon, 1945) between the original data and the harmonized data SDI values yielded a significant difference (*p*-value ≤0.01). In this comparison, the null hypothesis is that the difference of the original and harmonized SDIs has zero median. From this subject level analysis, it is clear that the harmonization has a 1) positive effect on the ability of the rater to be self consistent across the different scanners when delineating and 2) the original data results are in line with inter-scanner SDI and the harmonized results are close to reported intra-scanner SDI results. See Sec. 5.1 for a more complete discussion of these results.

**Table 3:** Intra-rater SDI comparing the manual delineations on the original data (OS1 vs. OS2) and harmonized data (HS1 vs. HS2). We report the range, median (Med.), mean, and standard deviation (SD) across the ten PwMS. There is a statistically significant difference (*p*-value ≤0.01), see the text for complete details. **Key:** Orig. - original data; Harm. - harmonized data.

	Range	Med.	**Mean(**±**SD)**
**Orig.**	[0.068, 0.667]	0.537	0.487(±0.187)
**Harm.**	[0.265, 0.814]	0.722	0.679(±0.166)

**Fig. 5:**
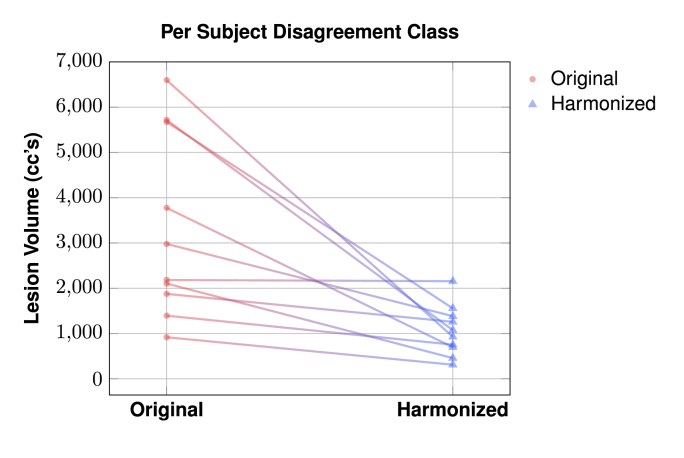
Shown for each subject is the volume of the Disagreement Class on both scanners as identified by the human delineator on the original and harmonized data. The lines connecting the two columns denote subject correspondence between the original and harmonized data—all of which decline.

For each of the ten PwMS we use the Disagreement Class to explore the disagreement between the delineations of the scanners, both original and harmonized. We plot the Disagreement volume in Fig. 5. It is the volume of lesions that have been identified in one subject's scans but not in the paired scan—i.e., the sum of lesion volume in a subject's scan from Scanner #1 that does not have any overlap in the corresponding Scanner #2 delineations and vice versa. We plot these volumes for all ten subjects in both the original and harmonized scenarios. We also draw a line, in Fig. 5, connecting the original result to its corresponding harmonized result. For all ten subjects, the volume of lesions in the Disagreement Class decreases between the original and harmonized scenarios. A two-sided paired Wilcoxon signed rank sum test between the original and harmonized volume of the Disagreement Class is significant (*p*-value ≤0.01). The 95 % confidence interval on the median of the difference is [3494, 9271] (Bauer, 1972; Hollander and Wolfe, 1973, pp. 68–75). Fig. 5 and the analysis show a dramatic and statistically significant improvement in reducing the disagreement after harmonization. Extended results for the subject level analysis are included in Appendix B.1.

### 4.2 Lesion & class analysis

We perform an analysis on a per lesion and per class basis between OS1 and OS2 in comparison to HS1 and HS2; for this we used the Condensed Nascimento Taxonomy (CNT) to categorize each lesion. We first present the SDI for the Agreement and Checkered Classes in Table 4. A two-sided Wilcoxon signed rank sum test for the Agreement Class between the original and the harmonized SDI values was statistically significant (*p*-value ≤0.01). A similar analysis of the Checkered Class was statistically significant (*p*-value ≤0.01).

**Table 4:** Intra-rater SDI comparing the manual delineations on the original (Orig.) and harmonized (Harm.) data on a per lesion basis for the Agreement and Checkered Classes. We report the range, median (Med.), mean, and standard deviation (SD).

	Agreement Class		
	**Range**	**Med.**	**Mean(**±**SD)**
**Orig.**	[0.008, 0.866]	0.467	0.450(±0.195)
**Harm.**	[0.028, 1.000]	0.608	0.569(±0.212)
	**Checkered Class**		
	**Range**	**Med.**	**Mean(**±**SD)**
**Orig.**	[0.000,^†^ 0.762]	0.038	0.158(±0.214)
**Harm.**	[0.000,^‡^ 0.874]	0.366	0.362(±0.276)

^†^ the value is 3.0 × 10^−5^.

^‡^ the value is 2.0 × 10^−4^.

**Fig. 6:**
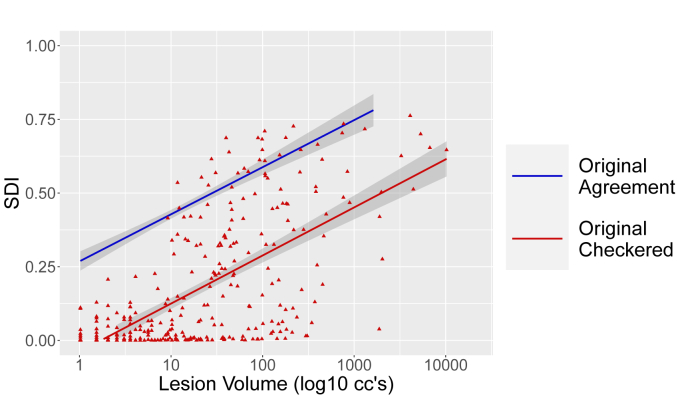
Shown on a per lesion basis is the Agreement and Checkered Classes between the original scanner data. The colored lines correspond to linear fits for the classes, with the shaded region around each line being the 95 % confidence interval for the linear fit.

**Fig. 7:**
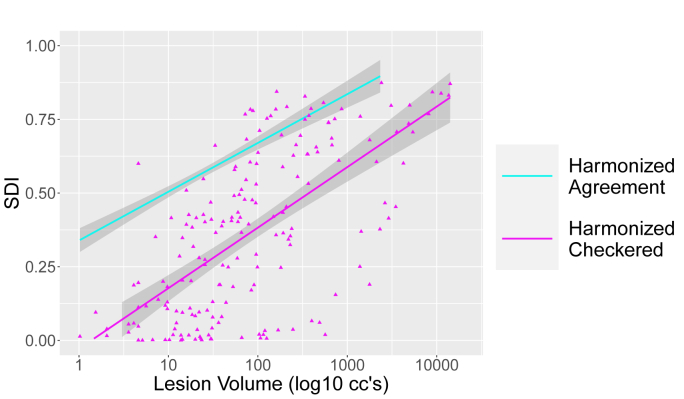
Shown on a per lesion basis is the Agreement and Checkered Classes between the harmonized scanner data. The colored lines correspond to linear fits for the classes, with the shaded region around each line being the 95 % confidence interval for the linear fit.

**Fig. 8:**
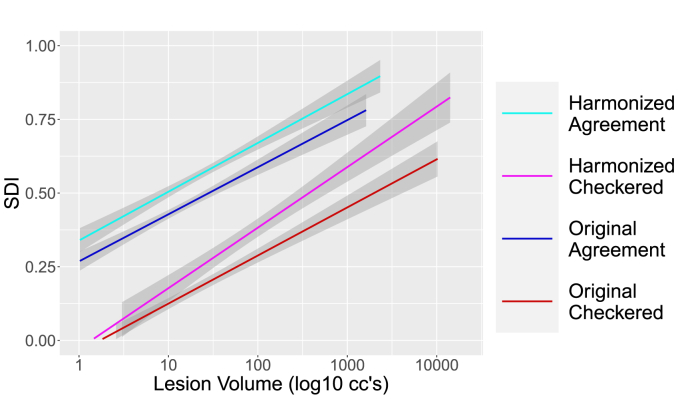
Shown are the linear fits to the Agreement and Checkered classes on the original and harmonized scanner data. We observe that the linear fits for both classes are higher on the harmonized data. See Fig. 6, Fig. 7 for the original data points.

In Fig. 6, we plot the lesion volume and the SDI for lesions in the original data—with different colors and markers for both classes—with the volume (*x*-axis) on a log scale. Fig. 6 also includes a least square linear regression for both of the classes, and the 95 % confidence interval for the regression is shown as the gray shaded region around the linear fit. Fig. 7 features the same style plot as Fig. 6, for the harmonized data. We do not include the Disagreement class in either Fig. 6 or 7, as the lesions in the Disagreement class all have an SDI of 0 and would just cover a span of the *x*-axis.To compare the linear fits for the two classes between the original and harmonized data, we replot the four linear fits (and their 95 % confidence intervals) in Fig. 8 keeping the same colors for the linear fits as used in Fig. 6, Fig. 7. In simple terms, the linear fits for the harmonized data are always above the original data, which tells us that across the full spectrum of lesion volumes the SDI is consistently higher in the harmonized data. The 95 % confidence intervals having only a tiny overlap across the range of volumes is also reassuring that these results are meaningful. We confirm this with a multivariate analysis of variance (MANOVA) test (Warne, 2010), which is included in Appendix B.2. The MANOVA establishes that there is a significant difference between the Agreement Class for the original and harmonized data and also a significant difference between the Checkered Class for the original and harmonized data. A complete discussion of these and other per lesion analyses results is in Sec. 5.2.

**Fig. 9:**
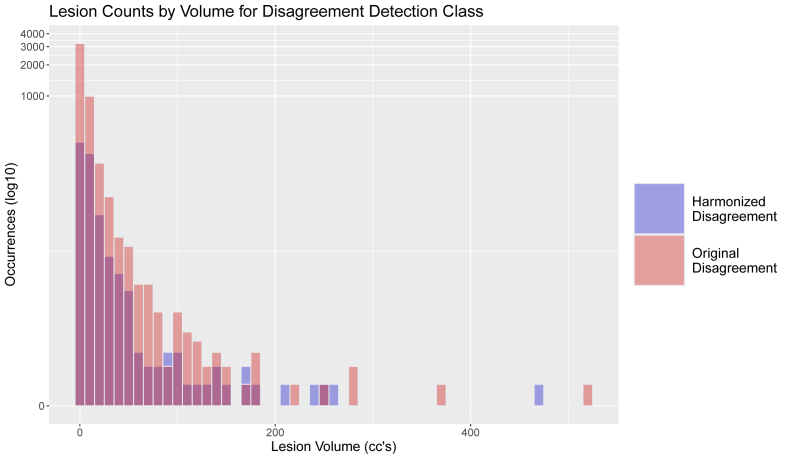
Histogram of the intra-rater Disagreement Class on the original data and the harmonized data. The original data Disagreement Class has 4700 lesions, whereas the harmonized data Disagreement Class has 793 lesions.

**Fig. 10:**
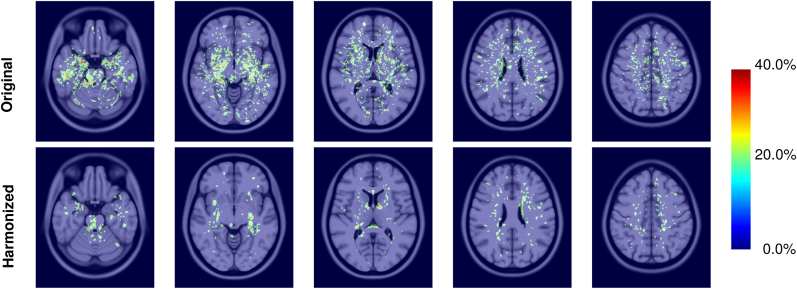
Shown from left to right are axial slices from the MNI atlas overlaid with an 8.8 mm thin-slab maximum intensity projection (TS-MIP) of the heat map for the Disagreement Class. The top row shows the Disagreement Class resulting from the Original data, while the bottom row shows the Disagreement Class resulting from the Harmonized data.

Next, we compare the Disagreement Class for the original and harmonized data. In Fig. 9, we present histograms of the volume of the lesions in the Disagreement Class. Recall that these are lesions that were identified in the manual delineations of one scanner but not the other, which means that by definition they have an SDI of 0 and thus we only perform analyses on the volumes. Due to the disparity in the number of small identified lesions, we use a log scale for the *y*-axis. The original data Disagreement Class has 4700 lesions, while the harmonized data Disagreement Class has 793 lesions. The statistics for these lesions are reported in Table 5. The mean and median for the original data are lower than the harmonized data, which is due to the enormous number of small lesions in the original delineations. We note that for almost every bin in the histograms of Fig. 9, the harmonized data lesion disagreement count is lower than the original data lesion disagreement count. A Mann-Whitney *U* test (Mann and Whitney, 1947) between the volume of the lesions for the original and harmonized data in the Disagreement Class is significant (*p*-value ≤0.01, with *W* = 2, 413, 310, and *η*^2^ = 0.032). Extended results for the lesion and class level analysis are included in Appendix B.2. As our data are processed in a common isotropic MNI template, we can create heat maps for the different classes, and explore any spatial bias in those same classes. We are motivated to explore this as Bozsik et al. (2022) observed a spatial bias to the inconsistency of their manual delineators, with the lesions in the optic nerve region being the most difficult to reliably identify. Within our heat maps, a value of 100.0 % would mean that a particular voxel saw disagreement between the original and harmonized data across all ten subjects. As the heat maps are sparse we create thin slab maximum intensity projection (TS-MIP) images (Napel et al., 1993), where the TS-MIP images are created as the maximum intensity over eleven adjacent 0.8 mm axial slices. We display these TS-MIP images overlaid on the axial slice of the MNI atlas that corresponds to the *central slice* of the eleven adjacent slices that generated the TS-MIP image. In Fig. 10, we display five of these TS-MIP images for the Disagreement Class of the original (top row) and harmonized (bottom row) data. Reviewing both rows of Fig. 10 suggests that for our data the disagreement is spatially diffuse and the improvement between the original and harmonized data is quite stark.

**Table 5:** For the Disagreement Class of the original (Orig.) and harmonized (Harm.) data, we report the range, median (Med.), mean, and standard deviation (SD) of the volumes (cc's) across all identified lesions.

	Range	Med.	**Mean(**±**SD)**
**Orig.**	[0.512, 516.096]	2.560	7.068(±17.953)
**Harm.**	[0.512, 471.552]	6.144	13.337(±30.445)

## 5 Discussion and conclusions

### 5.1 Global analysis

**Fig. B.1:**
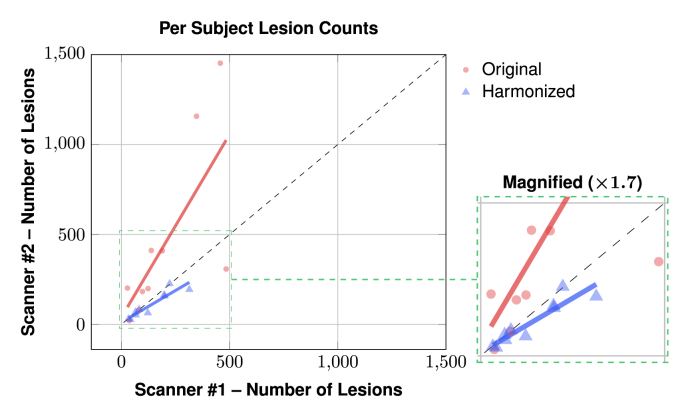
Shown for each subject are the lesion counts on both scanners as identified by the human delineator on the original and harmonized data. The lines correspond to linear fits of the original and harmonized data. The 45° line of unity would be the point where the lesion counts at both scanners are the same. The green box is shown beneath the legend with a magnification factor of × 1.7.

**Fig. B.2:**
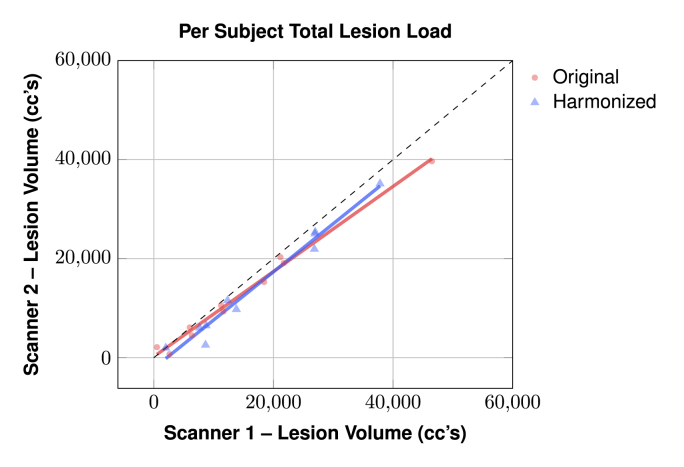
Shown for each subject are the lesion load (lesion volume) on both scanners as identified by the human delineator on the original and harmonized data. The lines correspond to linear fits of the data with matching colors. The 45° line of unity would be the point where the volumes identified for both scanners are the same.

Our mean SDI of 0.487 on the original data and 0.679 on the harmonized data, show the impact of the statistically significant improvement—see the subject level analysis in Table 3 for details. This improvement in consistency can also be seen in looking at the per subject lesion counts (see Fig. B1). Figure B2 presented the total lesion load (total lesion volume) across the ten subjects as delineated on the four versions of the data. The harmonized delineations had a consistent bias, while the original delineations had a bias that was lesion volume dependent. In Fig. 5, we could see a consistent statistically significant decrease in the lesion volume of the Disagreement Class.

With regards to the quality of our manual delineations, the 2008 MICCAI MS Lesion challenge (Styner et al., 2008) noted an SDI of 0.250, when comparing two manual delineators across ten subject scans. Our reported SDIs (see Table 3) are clearly better than this for both our original and harmonized delineations. This is encouraging and gives some confidence in the quality of the underlying delineations. However, the SDI reported in the 2008 MICCAI MS Lesion challenge (Styner et al., 2008) was an inter-rater comparison across scanners—thus inter-rater intra-scanner—whereas our results are an intra-rater inter-scanner comparison. The SDI will also depend on the lesion load of the subjects. Zijdenbos et al. (1994) reports a mean intra-rater SDI of 0.775 (two raters on four subjects), when restricted to the same scanner the intra-rater SDI increases to 0.789 (based on three subjects). Our harmonized data is close to this—our mean of 0.679 is less than a standard deviation (±0.166) from these quantities—but below them. Given the difference in cohort size (ten for us and three for Zijdenbos et al. (1994)) it is hard to draw definitive conclusions. We note that Egger et al. (2017) reported an inter-rater SDI of 0.66 on a cohort of 50 subjects. Based on this, we believe that our intra-rater performance is acceptable; more importantly it is clearly improved between the original and harmonized data. This is a key point that we wish to emphasize: *Across a broad collection of measures the harmonized delineation agreement is a statistically significant improvement on the original delineation agreement.*

### 5.2 Class analysis

We know that there is improvement in the agreement in going from the original to the harmonized data. Our Condensed Nascimento Taxonomy (CNT) allows us to go deeper, and tease apart the improvement in the delineations. This is done by exploring the different categories of lesions and examining their performance on both the original and harmonized data. In Table 4, we examined the Agreement and Checkered Classes, both of which improved from original to harmonized delineations. Our plots of volume against SDI, shown in Fig. 6, Fig. 7, with the corresponding class-wise linear fits allow us to see the differences in the performance of the two classes. Moreover, the combination of the linear fits on a single plot in Fig. 8 provides easy demonstration of the improvement after harmonization. Statistical testing, in the form of a MANOVA, confirms the significance of the difference. The Disagreement Class also sees a significant change. The total number of lesions in the Disagreement Class and the total volume of those lesions decreases. However, on a per lesion basis (from Table 4) we see that the mean volume of these lesions increases. We attribute this disparity to the large number of small lesions in the original delineations that pull the mean volume of such lesions lower. For reference, Fig. 9 displays a histogram of the lesion volumes in the Disagreement Class. The CNT is quite informative in this setting as it allows us to see that the improvement in the delineations between the original and harmonized data can be seen in all three classes and across the full range of lesion volumes.

### 5.3 Importance

An important point about these analyses and manual delineations of WMLs has been neglected up to this point. The goal of WML delineation (manual or automatic) is to quantify the current state of MS in a particular subject and hopefully be able to use these image based biomarkers to better understand the course of disease and the impact of therapeutics. As we can readily see in Sec. 4.1 there is a large variation in the delineations prior to harmonization (see also Sec. B.1 and Fig. B2). There are numerous studies that have used some combination of manual, semi-automated, or automated delineations of WMLs for various analytic purposes (van Walderveen et al., 1995; Schreiber et al., 2001; Chard et al., 2003; Quarantelli et al., 2003; Rovaris et al., 2010; Stankiewicz et al., 2011). None of which have conclusively demonstrated what cilinicians have long hypothesized: “*That disease state and lesion load are correlated.*” Given the presented work it seems reasonable to ask if the disease signal expressed in the MR images was simply being dominated by noise that can be mitigated by harmonization. This is a tantilizing thought, but one that is beyond the scope of this work.

### 5.4 Future work

An aspect that this paper did not cover is demonstration of improved DL performance given the improved delineations. We would expect the improvement to be two-fold: 1) segmentations with better agreement with manual delineations on harmonized data; 2) faster convergence of the deep network because of the improved consistency of the manual delineations and harmonized images. Of course it remains to be seen if this is true, however it does intuitively follow.

We have used the SDI in combination with object volumes for our CNT analyses. We could have also used other statistics to explore the intra-rater behavior across the harmonization barrier; in particular it might be interesting to consider a multi-dimensional representation (similar to Commowick et al. (2018)) for each of the CNT classes.

There are many aspects that influence the ability of manual delineators to identify objects in MRIs. These include, but are not limited to, underlying resolution (ie., spatial separation of data), sharpness, contrast, and noise. A goal of harmonization is to make these properties more consistent across scans. It would be desirable to tease apart both the effect of these different factors on delineations and how they are altered by the harmonization process. This is a definite avenue for continued research.

Another open question that should be resolved in future work, is the ability of these improved delineations to identify meaningful trends related to disease progression, be it on the cohort or subject specific level.

## Ethics statement

This study was performed in accordance with the Nuremberg Code and was approved by the Institutional Review Board of the Johns Hopkins Medical School. All participants provided written informed consent to participate in this study. Consent to publish identifying details was not acquired as the data for the study was collected retrospectively.

## CRediT authorship contribution statement

**Aaron Carass:** Writing – review & editing, Writing – original draft, Software, Methodology, Data curation, Conceptualization. **Danielle Greenman:** Writing – review & editing, Resources. **Blake E. Dewey:** Software, Data curation. **Peter A. Calabresi:** Writing – review & editing, Writing – original draft, Supervision, Resources, Project administration, Funding acquisition. **Jerry L. Prince:** Writing – review & editing, Supervision, Resources, Project administration, Funding acquisition. **Dzung L. Pham:** Writing – review & editing, Supervision, Resources, Project administration, Funding acquisition.

## Declaration of competing interest

The authors declare that they have no known competing financial interests or personal relationships that could have appeared to influence the work reported in this paper.

## Data Availability

The authors do not have permission to share data.

## Appendix

### A Lesion Protocol

The following protocol was used in the creation of the MS lesion masks, which were created in our 1 mm isotropic MNI space.

1. Review the possibilities for presentation of MS lesions in brain scans, an excellent resource is Sahraian and Radue (2007). It is also a good idea to familiarize yourself with the paint and mask functions in MIPAV before you begin, although this protocol description can serve as a basic guide.
2. Open MIPAV. If you have not done so in the past, add the Paint toolbar to the interface (Toolbars > Paint toolbar). The Image toolbar should be present by default; if not, go to Toolbars > Image toolbar.
3. Open two copies of the FLAIR scan and one copy each of the *T*_1_ − *w*, *T*_2_ − *w*, and PD-w scans (File > Open image (A) from disk > select files > Open). These should be appropriately co-registered in the axial view with identical slice thickness and field of view values before beginning this process.
4. Click on the *T*_1_ − *w* scan to select it, then click on the *WL* button on the MIPAV toolbar to bring up the Level and Window adjuster. This should automatically result in a reasonable tissue contrast for viewing potential lesions on the *T*_1_ − *w*. If the contrast is inadequate, change the window and level settings. Close the tool when you are satisfied with the contrast.
5. Enlarge the FLAIR images two times using the magnifying glass + button on the Image toolbar for a total magnification of 3×. Arrange the images on your display with the two FLAIR copies next to each other. The other images are kept at their original size, unless needed for further clarification in which case they are magnified as needed.
6. Link the scans together by first clicking on the *Sync slice number* button on the Image toolbar (two arrows one pointing left and the other pointing right). Then click on each scan and select the *Link images* button (broken links next to *Sync slice number* button; the broken links change to an intact link when activated). This will ensure that all of the scans stay on the same slice as the FLAIR while you work. Click on one of the scans, then scroll up and down while looking from side to side over the images to verify proper registration and check for image processing errors (e.g., missing pieces of brain).
7. For noisy images the delineation is completed entirely by hand. For low noise images, the following steps are used to complete the delineation:
  (a) Select one of the FLAIR scans, then click on the *Paint Grow* button (looks like a paint bucket) on the Paint toolbar to open the intensity and connectivity-based paint mask generator.
  (b) Open the Paint Power Tools plugin. The icon (lightbulb) should be at the right end of the Paint toolbar. Look at the *Threshold* section. Find the maximum intensity value present in the scan by observing the number in the right-hand box (upper threshold).
  (c) Look at the Paint Grow tool. Find the section marked *Set maximum slider values.* Change the maximum slider values in the paint mask generator to reflect the maximum intensity in the scan, and click *Set.*
  (d) Choose a lesion with well-defined borders and strong hyperintensity on the FLAIR scan. Click on the most hyperintense area in the lesion.
  (e) Move the second slider (Delta below selected voxel intensity) to the right until it encompasses most of the lesioned tissue.
  (f) Scroll up and down through the image to ensure that the selection is limited to the lesioned area and does not include hyperintensities due to noise or artifact. If non-lesioned tissue is included, move the slider back to the left until this tissue is deselected.
  (g) Move the first slider (Delta above selected voxel intensity) to the right to ensure that all voxels of higher intensity in the lesion are selected.
  (h) Repeat this process until all well-defined lesions in the FLAIR scan have been selected, remembering to scroll up and down frequently to prevent masking of non-lesioned tissue. In general, this process will result in a rough draft of a lesion mask.

**Do not** use this process for any area that is affected by scan artifact or for any hyperintensity that is not clearly a lesion. Investigate questionable areas during the later stages of the delineation process.

If a decision is to be made between fully encompassing a lesion and additional non-lesioned tissue or partially covering a lesion without extraneous tissue, choose the latter option. It tends to be easier, within MIPAV, to add to a mask than subtract from it.

(i) When you are satisfied with your rough mask, save it as an unsigned byte mask (VOI > Paint conversion > Paint to Unsigned byte mask). This will give you the binary mask data you have generated thus far. When the mask image appears, go to the File menu and choose *Save image as.* Enter the file name and desired extension, then click *Save.*
(j) Close the binary mask and the Paint Grow tool.

8. To begin, move your pointer over the area around the edge of a lesion, hold the mouse button down, and notice the intensity difference between the interior and exterior of the lesion. Record the intensity value for the area at the edge of the lesion.

Because many MS lesions are found in close proximity to the ventricles, it is useful to start in the middle slice in the axial view. Delineate from the middle axial slice to the superior aspect of the brain, scroll down to check your work, and then delineate from the middle to the inferior view.

9. On the Paint Power Tools interface, click the box next to *Threshold.* Enter the intensity value for the outside edge of the lesion in the first box; this will restrict your paint to voxels between that intensity (lower threshold) value and the value listed in the box to the right (upper threshold).

There is no need to change the value in the right box unless you are delineating lacunes. In that case, you should set the left box to the lowest possible value, and change the upper threshold to the highest value found on the edge of the lacune.

10. Click on the paintbrush icon on the paint toolbar. Paint around the edge of the lesion to test your threshold. You may need to paint and erase (paint = left mouse button, erase = right mouse button) the first time you do it, and then the threshold should be activated. You may also need to adjust the lower threshold value (left box). If too many voxels are being excluded from the lesion mask, lower the threshold value for a more inclusive range. If too many voxels are being included, increase the threshold value.
11. If you wish, you can change the paintbrush size by clicking on the drop-down menu in the center of the Paint toolbar and selecting one of the options.

You may also customize your paintbrush options by clicking on the *Paint brush editor* button (looks like a group of paintbrushes) to the right of this menu. This will open a grid size selector that allows you to specify the width and height of the grid for your paintbrush in pixels (default is 12 × 12). Click *OK*, and the grid will open. Draw the shape you want for your paintbrush, then go to the “Grid options” menu to save (Grid options > Save paint brush > input file name > Save). Your custom paintbrush will appear in the menu the next time MIPAV is opened, so restart the program if you want to use it immediately.

12. As you move to different slices, you may need to readjust the lower threshold. Not all of the lesion edges have the same intensity value, and intensities often differ between lesions at the anterior vs posterior areas of the same slice.
13. During this process, it is extremely important to scroll up and down frequently in order to get a sense of each lesion's shape and ensure mask continuity. For every hyperintensity identified, scrolling up and down can also help to rule out false positives. Be sure to look at the other scans, particularly the *T*_2_ − *w*, in order to verify that what you are selecting is a lesion.

In some cases, a lesion may be much more readily visible on the *T*_2_ − *w* scan. If this occurs, it is possible to delineate that portion directly on the *T*_2_ − *w* scan and add this small mask area to your FLAIR mask. This is particularly relevant when the FLAIR image contains a great deal of artifacts. If you cannot adequately capture the lesion on the FLAIR, use the *T*_2_ − *w*.

14. When debating what to include in the mask, keep these things in mind.
  (a) Lesions usually have rounded or smoothed edges.
  (b) Lesions appear distinctly hyper- or hypo-intense when compared with surrounding tissue (usually hyperintense on FLAIR, PD-w, and *T*_2_ − *w* scans, and hypointense on *T*_1_ − *w*),
  (c) Lesions will usually be found near the ventricles, in the corpus callosum, or in the deep white matter, though juxtacortical lesions are not uncommon.
  (d) Lesions may appear in the cerebellum, brainstem, temporal lobes, or basal ganglia at a lower intensity relative to the majority of the lesions. It is especially important to use information from the other scans when attempting to detect and delineate lesions in these areas.
  (e) Include white matter encompassed by closed, well-defined clusters of lesions. Do not include internal white matter if the cluster is open.
  (f) Include all CSF inside lacunes.
  (g) If a lesion is adjacent to clearly hyperintense areas near the ventricles, and you can confirm that these areas appear damaged in the *T*_1_ − *w* scan, include them in the mask. Lesioned tissue bordering the ventricles looks ragged and dark on *T*_1_ − *w* scans.
  (h) Do not include diffusely abnormal white matter (DAWM) in the masks. The intensity of DAWM is between normal white matter and lesioned tissue on FLAIR. DAWM looks mottled on *T*_1_ − *w*, may radiate outward like a halo from a focal lesion, and is usually found around the ventricles.
15. Save your work frequently or use the automatic save function in the Paint Power Tools interface. Check the box next to *Auto* save under *Misc.*, then set the number in the box to reflect how often you want the mask to be automatically saved (default is 10 min).
16. For some lesions, you may need to turn the paint threshold off and use the standard paint option, which will not restrict your paint to any specific intensity values. To do this, simply uncheck the box next to *Threshold.*
17. When you have finished delineating the lower portions of the brain, go back through the entire scan and check your work against the other images, focusing specifically on any areas that may have been difficult to verify as lesions. Edit as necessary.
18. Save your final mask.
19. To load a mask that you have worked on previously, select the FLAIR scan, then click on the second button from the left on the paint toolbar (appears to be a folder opening with a four-square gradient in front of it). Choose your mask file, click *Open*, and your mask will be loaded over the FLAIR.
20. If you would like to edit your mask after opening it from a saved file, open the Power Paint Tools, click on *Mask to Paint* under the *Import/Export* section at the bottom of the interface, and continue working.

### B Extended Results

#### B.1 Subject Level Analysis

For the subject level analysis comparing the SDI between the original data and harmonized data, we compute the he 95 % confidence interval on the median of the difference as [0.160, 0.223] (Bauer, 1972; Hollander and Wolfe, 1973, pp. 68–75).

We next report on the total lesion load and total number of lesions delineated in the original and harmonized data. We report these quantities by plotting the quantity assessed for Scanner #1 against the same quantity assessed for Scanner #2. In Fig. B1 we show the lesion count per subject for both the original and harmonized data analysis. The plot also includes the 45° (line of unity)—shown as dashes—which is the ideal line for the data to lie on; it represents the line upon which the total number of lesions agrees for a particular subject and scanner pair. In addition to the data points for the ten subjects, we include a least-squares linear regression to the data for both the original and harmonized delineations (with matching colors). The original data's linear fit has an adjusted *R*-squared of 0.466, and the harmonized data's linear fit has an adjusted *R*-squared of 0.838. Figure B1 also includes a magnified portion of the plot, highlighting the greater consistency in the subject-wise lesion counts in the harmonized data.

A two-sided paired Wilcoxon signed rank sum test between the original data's lesion counts has a *p*-value of 0.257 with a null hypothesis that the difference of the lesion counts between OS1 and OS2 has zero median. We note the 95 % confidence interval on the median of the differences is [−73, 372]. The equivalent test on the difference of the lesion counts between HS1 and HS2 has a *p*-value of 0.481 with a confidence interval on the median of the differences being [−42, 129]. These results highlight a disparity in the total number of lesions identified in the original data; while the harmonized data is more consistent in our cohort.

The total lesion load (in cc's) for both the original and harmonized data is shown in Fig. B2. The plot also includes the line of unity and the least-squares linear regression to the data for both the original and harmonized delineations (with matching colors). The original data's linear fit has an adjusted *R*-squared of 0.988, and the harmonized data's linear fit has an adjusted *R*-squared of 0.970. A two-sided paired Wilcoxon signed rank sum test between the original data's total lesion load has a *p*-value of 0.684 with a null hypothesis that the difference of the lesion counts between OS1 and OS2 has zero median. We note the 95 % confidence interval on the median of the differences is [−17224, 21791]. The equivalent two-sided paired Wilcoxon signed rank sum test between the harmonized data total lesion load has a *p*-value of 0.393 with a null hypothesis that the difference of the total lesion loads between HS1 and HS2 has zero median. The null hypothesis cannot be rejected and we note the 95 % confidence interval on the median of the differences is [−18718, 29999].

#### B.2 Lesion & Class Analysis

When comparing the Agreement Class of OS1 and OS2 against the Agreement Class of HS1 and HS2, we compute the 95 % confidence interval on the median of the differences as [0.103, 0.151]. A similar analysis of the Checkered Classes gives a 95 % confidence interval on the median of the differences as [0.128, 0.248]. We note that we could not use a paired-style Wilcoxon test as the number of lesions in these classes is different.

We perform a multivariate analysis of variance (MANOVA) test (Warne, 2010) between the Agreement and Checkered classes of both the original and harmonized data. A MANOVA analyzes the differences among groups in an independent variable by considering multiple dependent variables, which reduces Type I error that would be inflated by performing univariate analyses. We regard the original and harmonized data as two groups and are interested in the volume and SDI of the associated lesions. In this case the volume and SDI are two dependent variables, and our hypothesis is that both together are affected by the harmonization. A MANOVA was used to handle inter-correlations between dependent variables, which could be a concern when considering SDI and volume. The model of (SDI, Volume) ∼ Status, where Status denotes original or harmonized data, was significant (*p*-value ≤0.01) based on a Pillai Trace Test (Pillai, 1955) of the MANOVA. Thus, the null hypothesis that the group mean vectors are the same for the two groups is rejected.

We perform two more MANOVAs, where the first has the two groups being the Agreement Class for the original and harmonized data and the second has the two groups being the Checkered Class of the original and harmonized data. For the Agreement Class, the MANOVA was significant (*p*-value ≤0.01) based on a Pillai Trace Test with the same model. The same analysis on the Checkered Class data was also significant (*p*-value ≤0.01) based on a Pillai Trace Test. A post-hoc analysis confirms that both lesion volume and SDI are significantly affected by harmonization; for brevity the complete details are not included.

## Footnotes

1 Available from https://mipav.cit.nih.gov/.
